# 3D Body Scanning–Derived Normative Values of Appendicular Circumferences: A Novel Tool for Sarcopenia Screening in Chinese Adults

**DOI:** 10.1002/jcsm.70135

**Published:** 2025-11-16

**Authors:** Huijing He, Qiaolu Cheng, Zhiyue Zhang, Yaoda Hu, Zhiming Lu, Wei Han, Ji Tu, Ang Li, Zhen Song, Yawen Liu, Tan Xu, Qing Chang, Qiong Ou, Hui Pan, Zichao Wang, Guangliang Shan

**Affiliations:** ^1^ Department of Epidemiology and Statistics Institute of Basic Medical Sciences, Chinese Academy of Medical Sciences & School of Basic Medicine, Peking Union Medical College Beijing China; ^2^ State Key Laboratory of Common Mechanism Research for Major Diseases Beijing China; ^3^ State Key Laboratory of Experimental Hematology, National Clinical Research Center for Blood Diseases, Haihe Laboratory of Cell Ecosystem, Institute of Hematology & Blood Diseases Hospital, Chinese Academy of Medical Sciences & Peking Union Medical College Tianjin China; ^4^ Tianjin Institutes of Health Science Tianjin China; ^5^ School of Public Health Jilin University Changchun China; ^6^ School of Public Health Jiangsu Key Laboratory of Preventive and Translational Medicine for Geriatric Diseases, MOE Key Laboratory of Geriatric Diseases and Immunology, Suzhou Medical College of Soochow University Suzhou China; ^7^ Shengjing Hospital China Medical University Shenyang China; ^8^ Sleep Center, Department of Pulmonary and Critical Care Medicine Guangdong Provincial People's Hospital (Guangdong Academy of Medical Sciences) Southern Medical University, Guangdong Provincial Geriatrics Institute Guangzhou China; ^9^ Department of Endocrinology Key Laboratory of Endocrinology of National Health Commission, Translation Medicine Centre, Peking Union Medical College Hospital, Peking Union Medical College, Chinese Academy of Medical Sciences Beijing China; ^10^ Key Laboratory of Endocrinology of National Health Commission, Department of Endocrinology, State Key Laboratory of Complex Severe and Rare Diseases, Peking Union Medical College Hospital, Chinese Academy of Medical Science and Peking Union Medical College Beijing China

**Keywords:** 3D scan, appendicular circumferences, body composition, muscle health, reference values, sarcopenia

## Abstract

**Background:**

Appendicular circumferences (ACs) are critical predictors of skeletal health, cardiovascular disease risk and mortality. Currently, comprehensive reference values and associated factors for thigh (TC), calf (CC), upper arm (UAC) and forearm (FC) circumferences remain unestablished in Chinese adults.

**Methods:**

This community‐based cross‐sectional study (China National Health Survey, April 2023–November 2024) enrolled 8915 adults (≥20 years) for reference value development and 10 632 for association analysis. ACs were measured via 3D scanning, body composition via bioelectrical impedance analysis and handgrip strength using a Jamar dynamometer. Sex‐specific centile curves (P2.5–P97.5) were generated using lambda‐mu‐sigma methods. Propensity score matching balanced age distributions for sex and menopause subgroup comparisons. Multivariate logistic regression was used to examine factors associated with low appendicular circumference (lowest 5th percentile).

**Results:**

Median (25th and 75th) values for men were TC: 59.27 cm (55.09, 64.18), CC: 39.20 cm (37.17, 41.59), UAC: 28.31 cm (26.75, 30.10) and FC: 27.02 cm (25.67, 28.29); for women, TC: 52.45 cm (49.50, 55.74), CC: 35.71 cm (33.95, 37.76), UAC: 27.71 cm (25.74, 30.10) and FC: 24.64 cm (23.33, 26.07). Age trajectories showed sex‐specific patterns: TC, CC and UAC peaked at 20–29 years with subsequent decline, while FC peaked at 40–49 years. BMI‐adjusted circumferences exhibited divergent aging trajectories by sex. Postmenopausal women had significantly lower appendicular skeletal muscle mass (ASM), appendicular skeletal muscle mass index (ASMI), handgrip strength and CC than age‐matched premenopausal women (all *p* values < 0.05). All ACs strongly correlated with muscle mass, fat mass and muscle strength (all *p* values < 0.001), with UAC and FC showing the strongest ASM and ASMI correlations in men (correlation coefficient: 0.757 and 0.735). Factors associated with low ACs included rural residence, lower education, low BMI, elevated body fat (positively linked to low CC, especially ≥ 60 years) and cardiometabolic disorders (diabetes, hypertension, hyperuricemia, dyslipidemia).

**Conclusions:**

This study establishes the first age‐ and sex‐stratified percentile references for ACs in Chinese adults. These results reveal significant sex disparities in absolute and BMI‐adjusted measures, providing essential tools for sarcopenia screening, lifestyle intervention evaluation and high‐risk population identification.

## 1 Introduction

Sarcopenia, characterized by the accelerated loss of skeletal muscle mass and function associated with advancing age, represents a significant public health challenge due to its association with adverse outcomes including falls, fractures and premature mortality [1]. Current epidemiological data indicate that this condition affects approximately 10%–16% of the elderly population worldwide [2]. Despite its clinical significance, muscle wasting remains underrecognized, with many individuals and healthcare providers perceiving it as an inevitable consequence of aging rather than a modifiable condition [3].

Limb girths, including thigh circumference (TC), calf circumference (CC), upper arm circumference (UAC) and forearm circumference (FC), have emerged as important anthropometric indicators for both sarcopenia and obesity phenotypes. TC not only reflects body muscle mass but also peripheral subcutaneous fat distribution [4], with numerous studies demonstrating its association with cardiovascular disease risk and mortality [5]. CC serves as a key parameter in sarcopenia screening, with the Asian Working Group for Sarcopenia (AWGS) recommending specific cutoff values (≤ 34 cm for males and ≤ 33 cm for females) for initial case identification [6]. UAC has shown strong correlations with muscle mass measurements [7] and has demonstrated good performance in identifying sarcopenia [8]. Recent evidence further suggests that incorporating arm circumference measurements into existing screening tools like SARC‐CalF can significantly improve diagnostic accuracy for sarcopenia identification [9]. However, despite the clinical utility of these measures, comprehensive reference ranges for TC, CC, UAC and FC remain undefined for the general Chinese adult population, creating a significant barrier to early identification of individuals at high risk of sarcopenia. Specifically, this gap critically limits the practical application of anthropometric screening in several ways: (1) hampers screening tool implementation: The absence of validated, population‐specific reference ranges for CC (and potentially other circumferences like TC, UAC, FC) casts uncertainty on the applicability of existing international or regional thresholds within diverse Chinese healthcare and community settings. This ambiguity hinders the confident adoption and standardization of these simple, cost‐effective screening methods, particularly in resource‐limited primary care or large‐scale community health initiatives and (2) limits clinical interpretation and early risk detection: Without established age‐ and sex‐ specific normative values, clinicians lack benchmarks to interpret individual measurements consistently. This impedes early identification of at‐risk individuals before functional decline or complications manifest, likely missing opportunities for timely preventive interventions for sarcopenia and related cardiometabolic risks.

The advent of 3D body scanning technology has revolutionized anthropometry by enabling rapid, noncontact capture of high‐precision whole‐body surface measurements [10]. Validation studies confirm its excellent accuracy for circumferential analysis [11], positioning it as an ideal tool for large‐scale community‐based sarcopenia screening initiatives. Although preliminary studies have utilized 3D body scanning to assess CC and other parameters in small sample sizes [12, 13]—exploring links between limb measurements, body shape characteristics and metabolic indicators—a significant gap persists in large‐scale population research examining appendicular circumferences in relation to body composition parameters and their metabolic determinants.

To address these critical knowledge gaps, utilizing representative large‐scale data from the China National Health Survey, this study aims to: (1) establish reference ranges; (2) assess associations with body composition and strength; and (3) identify associators of low circumference values. The findings from this investigation are expected to establish population‐specific reference standards that will directly inform the development of national screening protocols, enhance sarcopenia risk assessment in primary care settings, and provide evidence‐based data to support health policy frameworks—thus facilitating effective community‐based preventive management of high‐risk populations.

## 2 Methods and Materials

### 2.1 Data Source and Study Population

This study utilized data from the second phase (April 2023–November 2024) of the China National Health Survey (CNHS), a nationwide epidemiological investigation employing standardized protocols as previously described [14]. To ensure data integrity, comprehensive quality assurance measures were implemented: (1) interviewer training—all field staff completed training before data collection to standardize questionnaire administration and measurement techniques; (2) quality control—including double‐checking of questionnaires by QC supervisors, regular calibration/maintenance of anthropometric equipment per manufacturer guidelines and uniform deployment of instruments/reagents/laboratory methodologies across regional centres to ensure cross‐site comparability. The survey employed a multistage stratified cluster sampling design to recruit community‐dwelling adults aged ≥ 20 years who had resided in their local area for ≥ 5 years across four geographically representative regions: Guangdong Province (Southern China), Jiangsu Province (Central China), Tianjin Municipality (Northern China) and Liaoning Province (Northeastern China). Exclusion criteria comprised the following: (1) individuals with severe physical or mental health conditions; (2) pregnant or lactating women; (3) professional athletes or active military personnel; and (4) foreign nationals. From an initial pool of 22 959 eligible participants, comprehensive data collection was performed through: (i) standardized interviewer‐administered questionnaires assessing sociodemographic characteristics, health behaviours and medical history; (ii) detailed anthropometric evaluations; and (iii) collection of fasting venous blood samples after an 10‐h overnight fast for biochemical analyses including plasma glucose, serum lipid profiles and uric acid levels.

The study has been carried out in accordance with the Declaration of Helsinki. Ethical approval was obtained from the Bioethical Committee of the Institute of Basic Medical Sciences, Chinese Academy of Medical Sciences (No. 2022177). Written informed consent was obtained from the participants.

### 2.2 Appendicular Circumferences Measurement

Whole‐body anthropometric measurements were obtained using a semimobile Alpha3Ds 3D body scanning system (AlphaM4‐MS, China), featuring four synchronized scanning columns that simultaneously capture 360° body surface images from anterior, posterior and bilateral perspectives to generate complete 3D models within 13 s. The system's proprietary reconstruction software, compliant with ISO 20685 (General requirements for 3‐D scanning anthropometric methodologies) and ISO 8559 (Size designation of clothes—Part 1: anthropometric definitions for body measurement) standards, automatically extracts > 160 anthropometric parameters including stature, body mass, linear dimensions, circumferences and volumetric measurements. The precision and intraobserver reliability of the system were verified through repeated measurements, which showed high consistency with Coefficient Of Variation (CV) values consistently below 2%, attesting to the method's robustness. Prior to each scanning session, the system was calibrated according to manufacturer specifications to ensure measurement accuracy. Participants wore either standardized compression garments or minimal clothing (underwear only), along with swim caps during measurements, and were instructed to maintain a standardized posture: standing upright with feet positioned 30 cm apart on marked footplates, upper limbs slightly abducted (15° from torso) with elbows flexed at 20° and head oriented in the Frankfurt Horizontal Plane. During the 13‐s scanning procedure, individuals maintained breath‐hold at end‐expiration to minimize respiratory artefacts. Bilateral thigh, calf, upper arm and forearm circumferences were measured, with maximum values used for analysis to prevent muscle mass underestimation from asymmetry and aligning with established CC protocols [15].

### 2.3 Anthropometry, Body Composition and Handgrip Strength Measurement

Height measurements were obtained to the nearest 0.1 cm using a calibrated stadiometer (SECA, Germany), with participants standing barefoot in the Frankfurt plane position. Body weight was measured to the nearest 0.1 kg using a multifrequency segmental bioelectrical impedance analyser (BIA) (Tanita MC780MA, Japan), with participants wearing light clothing. This validated BIA system provided detailed body composition metrics, including quantification of fat and lean mass distribution across whole‐body, regional (trunk) and appendicular (arms and legs) compartments [16, 17]. Specifically, we extracted muscle mass, fat mass, fat mass percentage and fat‐free mass for bilateral upper and lower extremities. Appendicular skeletal muscle mass (ASM) was calculated as the sum of arm and leg skeletal muscle mass, with the ASM index derived by normalizing ASM to height squared (kg/m^2^). Handgrip strength (HGS) was assessed using a Jamar Hydraulic Hand Dynamometer (JAMAR, UK) following established protocols as previously described [18]. All participants were instructed to fast for ≥ 10 h and refrain from vigorous physical activity for 24 h prior to measurements, and all the measurements were performed in the morning.

### 2.4 Other Covariates

Body mass index (BMI) was calculated as weight (kg) divided by height squared (m^2^). Hypertension was defined as systolic blood pressure ≥ 140 mmHg, diastolic blood pressure ≥ 90 mmHg or self‐reported diagnosis. Diabetes diagnosis required either self‐reported history or fasting plasma glucose (FPG) > 7.0 mmol/L [19]. Dyslipidemia was classified according to the Chinese guidelines for adult dyslipidemia management [20], while hyperuricemia was defined by elevated serum uric acid levels or self‐reported diagnosis [21]. Smoking status was categorized into current, former or never. Current smoking was defined as smoking at least one cigarette per day for at least the past 6 months. Former was defined as having quit tobacco smoking for more than the 6 months preceding the survey. Alcohol drinking was defined as the consumption of at least 30 g of alcohol and had lasted at least 6 months [14]. Information on other diseases, such as cardiovascular diseases (CVDs), musculoskeletal disorders (MSDs, such as gout, arthritis diseases, spine conditions), cancers, etc., was collected based on self‐report.

### 2.5 Statistical Analysis

Participants were categorized into two analytical groups based on the study objectives: (1) a reference population (*n* = 8915) that excluded individuals lacking 3D scan data or with pre‐existing cardiovascular diseases, musculoskeletal disorders or cancers; and (2) an association analysis group (*n* = 10 632) consisting of all eligible participants with complete 3D scan measurements. The Dixon‐Reed method was applied to identify measurement outliers for all anthropometric variables [22].

We computed age‐ and sex‐specific reference values for appendicular circumferences (thigh, calf, upper arm and forearm) using both mean (standard deviation) and percentile distributions (2.5th, 5th, 25th, 50th, 75th, 95th and 97.5th). Growth patterns were modelled using the lambda‐mu‐sigma (LMS) method, which incorporates the following: 1) μ (median) representing central tendency; 2) σ (coefficient of variation) accounting for dispersion; and 3) λ (Box–Cox transformation parameter) adjusting for distributional skewness [18, 23]. Using the GAMLSS package in R (v4.3), cubic smoothing splines were employed to generate percentile curves for both raw and BMI‐adjusted measurements, with the latter calculated as absolute circumference values normalized to BMI.

Partial correlation analyses, adjusted for age and BMI and stratified by sex, examined relationships between circumferential measurements and body composition parameters. Propensity score matching balanced age distributions when comparing sex and menopause status differences in anthropometric measures. Wilcoxon signed‐rank tests were performed to compare the matched groups. Multivariate logistic regression models adjusted for covariates—including demographic characteristics (urban/rural residence, education), body composition indicators (BMI, body fat percentage, muscle mass), lifestyle factors (smoking, alcohol consumption, physical activity) and clinical profiles—identified factors associated with low appendicular circumferences (defined as < 5th percentile per age stratum), with sensitivity analyses excluding participants with chronic conditions in all age strata (cardiovascular diseases, musculoskeletal disorders or cancers).

**FIGURE 1:**
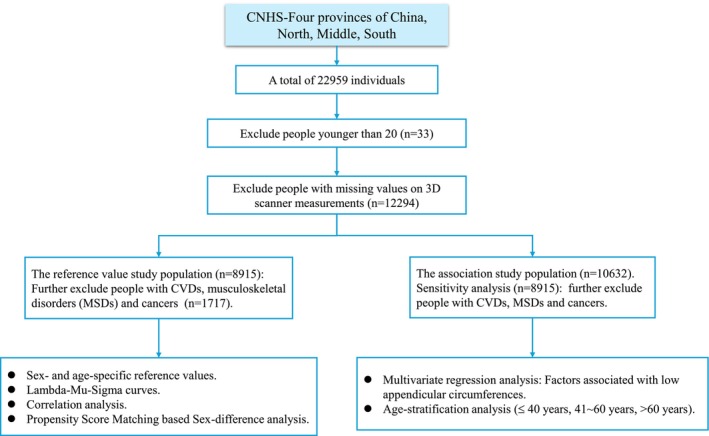
Flow chart of the study.

The flow chart of the study is presented in Figure 1. All statistical procedures were performed using SAS 9.4 (SAS Institute) and R 4.3, with two‐tailed *p* values < 0.05 considered statistically significant.

## 3 Results

### 3.1 Basic Characteristics of the Study Population

The demographic and anthropometric characteristics of the study population are presented in Supplementary Tables S1 and S2. The reference group comprised 8915 participants (mean age 53.44 ± 12.74 years; 39.4% male), with significant sex differences observed in all body composition parameters (all *p* < 0.001). The sample was predominantly composed of urban residents (62.45%), with 30.3% having attained college‐level education or higher (Supplementary Table S1). The association analysis group (*n* = 10 632), with a mean age of 54.77 ± 12.79, demonstrated similar baseline characteristics to the reference population (Supplementary Table S2).

### 3.2 Normative Values of Appendicular Circumferences

**FIGURE 2:**
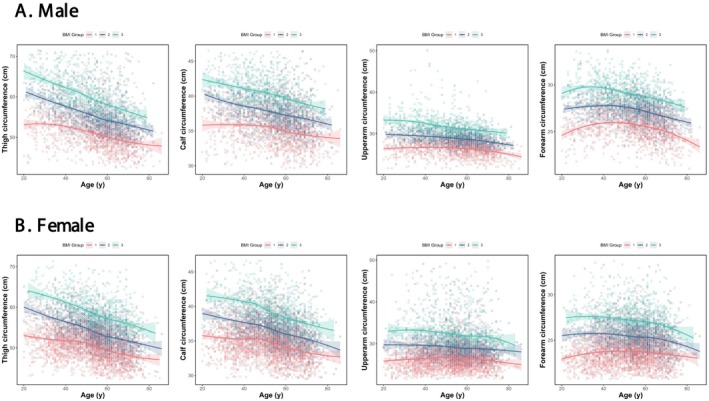
Age‐related trajectories of appendicular circumferences stratified by BMI categories. The cutoffs for BMI categories 1, 2 and 3 were < 24, 24~27.9 and ≥28 kg/m^2^.

The age‐stratified distributions of appendicular circumferences (thigh, calf, upper arm and forearm) across different BMI categories are presented in Figure 2. Detailed age‐ and sex‐specific reference values for thigh and calf circumferences are provided in Table 1, while corresponding data for upper arm and forearm circumferences are shown in Table 2.

**TABLE 1:** Normative values for 3D‐scan measured thigh and calf circumferences among unselected Chinese adults.

	Calf circumference								Thigh circumference						
	*n*	Mean	Std	5%	25%	50%	75%	95%	Mean	Std	5%	25%	50%	75%	95%
**Total**	8854	36.65	3.99	31.68	34.35	36.29	38.48	42.28	53.67	5.74	45.55	49.94	53.05	56.63	63.76
20—	397	38.69	5.47	32.08	35.57	37.89	40.61	46.31	57.48	6.82	47.49	52.53	56.95	61.76	70.33
30—	1126	37.45	3.81	32.35	34.93	37.16	39.46	43.00	55.89	6.06	47.47	51.62	55.00	59.54	66.66
40—	1679	37.56	3.93	32.86	35.20	37.15	39.30	43.03	55.45	5.47	47.86	51.61	54.81	58.44	65.08
50—	2623	36.75	4.05	32.07	34.60	36.37	38.42	41.93	53.56	5.14	46.70	50.35	53.03	56.18	62.23
60—	2271	35.66	3.17	30.94	33.71	35.47	37.41	40.37	51.86	5.03	44.57	48.62	51.45	54.60	60.55
70—	758	35.07	4.19	30.06	32.82	34.73	36.65	40.26	50.22	5.41	42.51	46.90	49.94	53.00	58.32
**Men**															
Overall	3501	37.51	4.10	32.22	35.20	37.22	39.39	43.32	54.90	6.43	46.08	50.69	54.03	58.18	66.42
20—	184	39.70	5.09	32.71	37.17	39.20	41.59	46.76	59.59	6.99	48.86	55.09	59.27	64.18	71.37
30—	435	38.74	3.90	33.30	36.72	38.57	40.45	44.26	58.53	6.38	49.15	54.38	57.81	62.01	70.73
40—	623	38.40	3.31	33.41	36.21	38.31	40.40	43.88	57.13	6.19	48.66	52.92	56.25	60.45	68.81
50—	984	37.78	4.78	32.68	35.47	37.32	39.35	43.00	54.74	5.71	47.49	50.98	54.02	57.31	64.61
60—	941	36.29	3.12	31.62	34.50	36.22	38.07	41.06	52.49	5.18	44.77	49.53	52.02	55.01	61.86
70—	334	35.64	3.63	30.77	33.44	35.49	37.40	40.84	50.68	6.21	42.71	47.52	50.17	53.02	59.40
**Women**															
Overall	5353	36.10	3.82	31.47	33.95	35.71	37.76	41.35	52.86	5.08	45.34	49.50	52.45	55.74	61.69
20—	213	37.82	5.66	32.05	34.88	36.86	39.34	44.67	55.65	6.13	47.44	51.58	54.82	59.21	66.94
30—	691	36.63	3.51	32.11	34.34	36.18	38.45	41.92	54.23	5.21	47.08	50.69	53.41	57.23	63.72
40—	1056	37.06	4.17	32.61	34.81	36.62	38.62	42.23	54.46	4.73	47.60	51.07	54.06	57.23	62.94
50—	1639	36.13	3.40	31.74	34.22	35.84	37.67	40.95	52.85	4.62	45.98	49.92	52.50	55.47	60.42
60—	1330	35.20	3.12	30.74	33.35	35.01	36.85	39.97	51.41	4.87	44.37	48.13	51.08	54.23	59.85
70—	424	34.62	4.53	29.77	32.46	34.24	35.89	39.21	49.86	4.66	42.47	46.68	49.80	52.96	57.98

**TABLE 2:** Normative values for 3D‐scan measured upper arm and forearm circumferences.

	Upper arm circumference								Forearm circumference						
	*n*	Mean	Std	5%	25%	50%	75%	95%	Mean	Std	5%	25%	50%	75%	95%
**Total**	8915	28.85	6.57	23.50	26.16	27.99	30.10	34.89	26.18	6.53	22.00	23.98	25.56	27.26	29.88
20—	399	28.58	5.54	22.13	25.38	28.2	30.92	35.21	25.96	5.56	21.37	23.42	25.47	27.90	30.90
30—	1132	28.96	6.00	23.01	26.03	28.19	30.71	35.83	26.31	6.84	21.60	23.74	25.70	27.57	30.56
40—	1692	29.47	7.43	23.83	26.59	28.40	30.69	35.85	26.61	7.22	22.18	24.20	25.97	27.79	30.26
50—	2645	29.11	6.86	23.96	26.38	28.21	30.15	34.93	26.37	6.75	22.37	24.13	25.70	27.35	29.74
60—	2282	28.39	5.69	23.64	26.11	27.65	29.53	33.80	25.83	5.33	22.13	24.06	25.42	26.86	28.99
70—	765	27.96	7.10	22.62	25.38	27.01	28.81	32.91	25.54	7.28	21.42	23.36	24.83	26.30	28.48
**Men**															
Overall	3510	28.72	4.69	24.31	26.75	28.31	30.10	33.21	27.25	4.48	23.90	25.67	27.02	28.29	30.47
20—	184	29.46	3.47	24.14	27.03	29.48	31.88	35.41	27.31	2.53	23.09	25.48	27.53	28.97	31.75
30—	435	29.51	3.54	24.91	27.48	29.05	31.17	34.77	27.87	5.15	24.50	26.30	27.55	29.00	31.38
40—	625	29.68	5.91	24.93	27.55	29.09	31.17	34.09	28.02	5.13	24.64	26.57	27.73	29.03	30.94
50—	990	29.02	5.88	24.83	27.00	28.51	30.13	32.91	27.58	5.02	24.33	25.98	27.21	28.50	30.39
60—	941	27.86	2.33	24.31	26.44	27.74	29.19	31.71	26.50	1.92	23.74	25.37	26.50	27.56	29.32
70—	335	27.04	4.12	23.16	25.39	26.85	28.06	30.97	26.13	5.85	22.49	24.36	25.59	26.89	28.53
**Women**															
Overall	5405	28.94	7.55	23.14	25.74	27.71	30.10	37.04	25.48	7.49	21.64	23.33	24.64	26.07	28.78
20—	215	27.82	6.75	21.64	24.37	26.76	30.15	34.58	24.81	7.00	20.74	22.52	23.91	25.67	28.54
30—	697	28.61	7.10	22.51	25.30	27.52	29.96	37.72	25.34	7.55	21.26	22.92	24.41	26.01	29.05
40—	1067	29.35	8.19	23.51	26.04	27.96	30.36	37.30	25.79	8.08	21.82	23.53	24.78	26.28	29.02
50—	1655	29.16	7.39	23.54	26.01	27.96	30.20	37.23	25.64	7.50	21.97	23.47	24.77	26.21	28.84
60—	1341	28.77	7.14	23.30	25.75	27.54	29.89	36.83	25.36	6.73	21.70	23.46	24.63	25.98	28.37
70—	430	28.67	8.69	22.37	25.37	27.23	29.55	35.22	25.08	8.20	20.96	22.87	23.97	25.46	28.26

**FIGURE 3:**
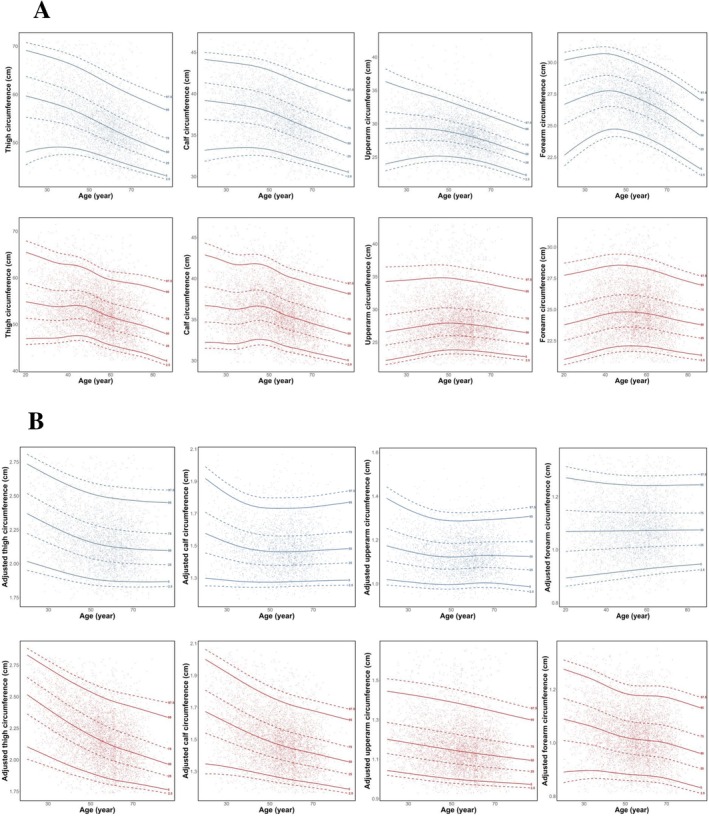
Lambda‐mu‐sigma centile curves for appendicular circumferences. (A) Sex‐specific calf,thigh, upper arm and forearm circumferences. (B) BMI‐adjusted sex‐specific calf, thigh, upper arm and forearm circumferences.

Median values (25th and 75th) for male participants were as follows: CC 39.20 cm (37.17, 41.59), TC 59.27 cm (55.09, 64.18), UAC 28.31 cm (26.75, 30.10) and FC 27.02 cm (25.67, 28.29). Female participants demonstrated smaller median values: CC 35.71 cm (33.95, 37.76), TC 52.45 cm (49.50, 55.74), UAC 27.71 cm (25.74, 30.10) and FC 24.64 cm (23.33, 26.07). Age‐related trajectories of both absolute and BMI‐adjusted appendicular circumferences are illustrated in Figure 3. Among male participants, thigh, calf and upper arm circumferences exhibited peak values in young adulthood (20–29 years) followed by progressive decline with advancing age. In contrast, forearm circumference demonstrated a different pattern, increasing until middle age (40–49 years) before declining. Female participants showed markedly different trajectories, particularly for arm circumferences (Figure 3A). Significant sex differences were observed in the aging patterns of BMI‐adjusted appendicular circumferences (Figure 3B).

Age‐matched comparisons revealed that male participants had significantly greater appendicular muscle mass but lower fat mass percentage compared to females (all *p* < 0.001). However, no statistically significant sex differences were observed in appendicular circumference measurements (Supplementary Figure S1). There are significant differences between postmenopausal and premenopausal women in ASM, ASMI, HGS and CC. Crucially, among women aged 45–55 years—representing the peak menopausal transition—postmenopausal women exhibited lower muscle mass (ASM/ASMI), reduced muscle strength and decreased CC (Supplementary Figure S2).

### 3.3 Correlations Between Appendicular Circumferences, Body Composition and Muscle Strength Indicators

**FIGURE 4:**
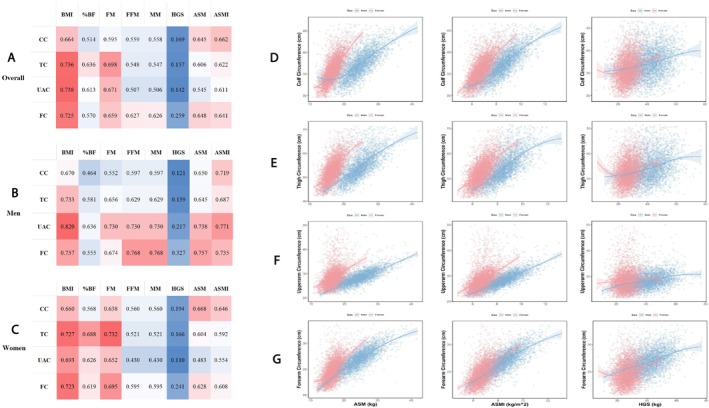
Correlation analyses between appendicular circumferences and body composition parameters. (A) Overall population (sex‐ and age‐adjusted). (B) Males (age‐adjusted). (C) Females (age‐adjusted). (D–G) Appendicular skeletal muscle mass, appendicular skeletal muscle index, and handgrip strength correlations with individual circumferences (CC, TC, UAC, FC). Abbreviations: %BF, body fat percentage; ASM, appendicular skeletal muscle mass; ASMI, appendicular skeletal muscle mass index; BMI, body mass index; CC, calf circumference; FC: forearm circumference; FFM, fat free mass; FM, fat mass; MM, muscle mass; HGS: hand grip strength; TC: thigh circumference; UAC: upper arm circumference.

Figure 4 illustrates the correlations between TC, CC, UAC and FC with body composition parameters and muscle strength. Overall, the four appendicular circumference indices demonstrated significant associations with BMI, adiposity‐related indices (including %BF and fat mass) and muscle mass indicators such as ASM and ASMI, with notable sex‐specific differences observed. Notably, the correlations of UAC and FC with ASM and ASMI were significantly stronger in males (with correlation coefficients of 0.757 and 0.735, respectively) compared to females (0.628 and 0.608). Additionally, across both sexes, ASM exhibited the highest correlation coefficient with FC (with a correlation coefficient of 0.648). The exact values for these correlations are shown in each cell in Figure 4 (A)‐(C). The correlation between TC, CC, UAC and FC with ASM, ASMI and HGS are further displayed using scatter plots and regression curves in Figure 4 (D)‐(G).

### 3.4 Factors Associated With Low Appendicular Circumferences

Analyses identifying factors associated with low TC, CC, UAC and FC are summarized in Table 3. Key findings for each circumference are detailed below.

**TABLE 3:** Factors associated with low appendicular circumferences.

	Low calf circumference				Low thigh circumference				Low upper arm circumference				Low forearm circumference			
	OR	95% CI		*P*	OR	95% CI		*P*	OR	95% CI		*PP*	OR	95% CI		*P*
**Urban**	Ref	NA		NA	Ref	NA		NA	Ref	NA		NA	Ref	NA		NA
**Rural**	1.452	1.121	1.880	0.005	1.112	0.839	1.474	0.460	0.568	0.426	0.758	<0.001	0.610	0.463	0.804	0.001
**Education**																
Illiterate	1.652	1.124	2.428	0.011	1.273	0.835	1.943	0.262	1.070	0.701	1.633	0.754	0.733	0.485	1.106	0.139
Elementary school	1.492	1.094	2.036	0.012	1.299	0.929	1.818	0.127	1.083	0.788	1.489	0.621	0.923	0.681	1.251	0.605
High school	0.995	0.724	1.367	0.973	1.083	0.781	1.502	0.633	0.943	0.696	1.277	0.705	0.907	0.678	1.213	0.510
College or above	Ref	NA		NA	Ref	NA		NA	Ref	NA		NA	Ref	NA		NA
BMI	0.466	0.422	0.513	<0.001	0.496	0.446	0.553	<0.001	0.571	0.515	0.632	<0.001	0.670	0.609	0.737	<0.001
%BF	1.062	1.030	1.095	<0.001	0.934	0.903	0.965	<0.001	0.925	0.895	0.956	<0.001	0.922	0.892	0.952	<0.001
Muscle mass	0.977	0.947	1.008	0.150	0.897	0.866	0.929	<0.001	0.887	0.857	0.919	<0.001	0.857	0.828	0.887	<0.001
**Smoking status**																
Never	Ref	NA		NA	Ref	NA		Ref	Ref	NA		NA	Ref	NA		NA
Former	1.780	1.069	2.963	0.027	1.491	0.866	2.568	0.150	1.455	0.844	2.507	0.177	3.045	1.843	5.031	<0.001
Current	2.440	1.720	3.462	<0.001	1.889	1.299	2.747	0.001	2.043	1.405	2.970	<0.001	2.852	1.961	4.148	<0.001
**Alcohol drinking**																
Never	Ref	NA		NA	Ref	NA		Ref	Ref	NA		NA	Ref	NA		NA
Former	0.939	0.521	1.693	0.834	0.821	0.437	1.541	0.540	0.948	0.503	1.784	0.868	0.676	0.366	1.250	0.212
Current	1.258	0.929	1.704	0.137	1.154	0.836	1.593	0.384	1.425	1.049	1.937	0.024	0.977	0.717	1.330	0.881
**Physical exercise**																
5~7 days per week	Ref	NA		NA	Ref	NA		Ref	Ref	NA		NA	Ref	NA		NA
3~4 days per week	0.951	0.630	1.434	0.809	0.943	0.607	1.463	0.793	0.941	0.631	1.402	0.764	0.629	0.412	0.960	0.032
1~2 days per week	0.920	0.612	1.384	0.691	1.109	0.729	1.689	0.629	0.797	0.526	1.207	0.284	0.857	0.580	1.265	0.437
≤3 days per month	0.965	0.626	1.486	0.870	0.836	0.514	1.358	0.469	0.933	0.596	1.460	0.761	0.652	0.411	1.036	0.070
Never	1.283	0.997	1.652	0.053	1.169	0.886	1.543	0.270	0.869	0.659	1.146	0.319	0.918	0.707	1.191	0.519
HTN	1.331	1.035	1.711	0.026	1.554	1.182	2.043	0.002	0.964	0.730	1.273	0.798	1.268	0.981	1.641	0.070
DM	1.874	1.355	2.593	<0.001	4.094	2.958	5.668	<0.001	1.759	1.226	2.522	0.002	0.864	0.585	1.275	0.460
HUA	1.214	0.916	1.609	0.178	1.411	1.038	1.917	0.028	1.303	0.963	1.763	0.086	1.282	0.964	1.704	0.088
Low HDL‐C	1.124	0.750	1.685	0.570	1.645	1.086	2.491	0.019	1.002	0.636	1.579	0.991	1.114	0.733	1.693	0.614
High non‐HDL‐C	1.379	1.034	1.840	0.029	1.347	0.973	1.865	0.072	1.109	0.800	1.537	0.536	1.078	0.793	1.464	0.633

Abbreviations: CI: confidence interval; DM: diabetes mellitus; HDL‐C: high‐density lipoprotein cholesterol; HTN: hypertension; HUA: hyperuricemia; OR: odds ratio.

Low CC: Significantly higher odds of low CC were observed in individuals residing in rural areas, those with lower educational attainment, lower BMI, higher %BF, a history of cigarette smoking or comorbidities including HTN, DM or elevated non‐HDL cholesterol.

Low TC: Factors significantly associated with low TC included lower BMI, lower %BF, lower muscle mass, current cigarette smoking and comorbidities such as HTN, DM, hyperuricemia and low HDL‐C.

Low UAC and FC: Risk factors for low UAC and FC were largely overlapping. Individuals living in urban areas, those with lower BMI, lower %BF, lower muscle mass or a history of (ever or current) cigarette smoking had significantly higher odds. Notably, DM was specifically associated with increased odds of low UAC.

In summary, lower BMI consistently predicted low values across all four circumferences (TC, CC, UAC, FC). Cigarette smoking was a strong risk factor: Both current and former smoking were associated with low CC, UAC and FC, while only current smoking was specifically linked to low TC. Metabolic comorbidities, particularly HTN and DM, were frequently associated with low circumferences (HTN and DM with low CC and TC; DM specifically with low UAC). Refer to Table 3 for the exact odds ratios (ORs) and 95% confidence intervals (CIs).

The age‐stratified association analyses are shown in Supplementary Table S3–Table S5. Associated factors for low appendicular circumferences among different age groups are similar, while people aged over 40 are more likely to be influenced by metabolic disorders such as DM and dyslipidemia. Supplementary Table S6 shows the results of the sensitivity analysis; the main results remain consistent with the primary association analysis.

## 4 Discussion

This study establishes, to our knowledge, the first comprehensive reference values for four key appendicular circumferences (thigh, calf, upper arm and forearm) in a general Chinese adult population spanning a broad age spectrum. Our findings delineate distinct sex‐ and age‐specific growth trajectories for these anthropometric parameters, providing novel insights into their developmental patterns across adulthood. Importantly, we observed significant sex‐based differences in both absolute circumference measurements and body composition parameters, while also elucidating key factors associated with reduced appendicular circumferences. These normative data and associated findings hold substantial clinical relevance, offering valuable tools for identifying high‐risk populations in sarcopenia screening and prevention programs.

Our findings demonstrate a consistent age‐related decline in absolute circumference measurements (thigh, calf, mid‐upper arm and forearm) commencing in middle age, with an earlier onset of decline observed in lower extremity measurements (thigh and CC) compared to upper extremities. Notably, sex‐specific patterns emerged when examining BMI‐adjusted values: Male participants maintained relatively stable adjusted circumference measurements (TC, CC, MUAC, FC) after middle age, whereas female participants showed progressive declines. This sex‐based difference in BMI‐normalized trajectories likely reflects fundamental differences in aging physiology between sexes. Specifically, postmenopausal women experience accelerated sarcopenic changes (as shown in supplementary Figure S2) potentially mediated by oestrogen decline and its catabolic effects on muscle tissue, while aging men tend to preserve central adiposity patterns that may partially mitigate circumference reductions. These findings align with established literature on sex differences in body composition changes during aging [24].

Current sarcopenia guidelines predominantly rely on CC as a screening indicator [25], while emerging evidence suggests clinical utility for other appendicular circumferences. Crucially, our results (Table 1/Figure 3) demonstrate significant age‐dependent variation in normative CC values. This finding contrasts with widely used nonstratified diagnostic criteria [6, 25] and importantly reveals that applying uniform cutoffs would require higher thresholds for younger adults than currently established. Such misalignment suggests potential underestimation of sarcopenia risk in younger populations with current criteria. Therefore, our provision of age‐ and sex‐specific CC reference ranges addresses a critical gap for precise risk assessment.

Furthermore, thigh (TC), upper arm (UAC) and forearm (FC) circumferences all exhibit strong correlations with muscle mass and body composition [26]. These measurements demonstrate comparable clinical associations with established muscle loss risk factors, supporting their utility as complementary screening targets—particularly when CC measurement is not available (e.g., oedema or amputation). Comprehensive characterization of these parameters across demographic strata enables a more holistic muscular health evaluation, facilitating early detection of sarcopenia.

Existing literature on cutoff values for appendicular circumferences in sarcopenia screening remains limited. Previous studies have primarily focused on specific population subgroups, with Li et al. [27] establishing optimal cutoff values for UAC (25.9 cm for males, 26.5 cm for females) and CC (33.7 cm for males, 33.0 cm for females) among Chinese community‐dwelling older adults—values consistently higher than the 5th percentile cutoffs identified in our study for individuals aged > 60 years. Gonzalez et al. [15] further contributed to this field by deriving CC cutoffs (among people with a BMI 18.5–24.9 kg/m^2^) from NHANES 1999–2006 data. Importantly, while CC has been incorporated into current screening guidelines, standardized reference ranges for thigh, upper arm and FCs remain undefined in general populations. This critical gap in knowledge hinders the development of more comprehensive sarcopenia screening approaches that could benefit from utilizing multiple anthropometric parameters.

When establishing reference values for appendicular circumferences, BMI stratification or normalization is critical to control for adiposity‐related confounding. This approach aligns with the conceptual framework of sarcopenic obesity [28], which emphasizes evaluating not only absolute measurements but also body composition heterogeneity (i.e., divergent muscle‐to‐fat ratios) within identical circumference ranges—particularly in older adults. Our findings demonstrate that BMI‐adjusted TC, CC, UAC and FC exhibit divergent age‐related trajectories versus absolute values, indicating sex‐ and age‐dependent variations in sarcopenic obesity risk.

Our study demonstrated significant correlations between appendicular circumferences and both ASM and ASMI, which are established indicators for sarcopenia screening [29]. The observed sex differences in these associations may be attributed to inherent variations in body composition between sexes [18], as evidenced by the distinct patterns of muscle mass and fat percentage distribution presented in our sex‐specific analysis. Specifically, the strongest correlation between FC and ASM/ASMI indicated potential clinical utility of FC as a proxy for ASM. Current sarcopenia screening protocols typically employ a sequential approach, initiating with muscle strength assessment followed by evaluation of muscle mass parameters (e.g., ASM and ASMI) when initial measurements fall below established thresholds [29]. Although FC has been relatively understudied and has not been widely implemented in community‐based sarcopenia screening programs, our findings reveal strong correlations between FC and both ASM and ASMI. These results suggest FC may possess potential utility as a screening metric for sarcopenia, particularly in male populations, warranting further investigation.

Muscle health is correlated with socio‐economic factors. Our previous study has revealed that social‐economic inequalities and early life malnutrition contribute to the low hand grip strength [30], suggesting that they are also risk factors for other indicators of sarcopenia. Our study revealed that people living in rural areas, having lower education levels, are more likely to have low CC, and lower educational levels are further associated with low MUAC and low FC. These findings align with existing literature on the social and structural determinants of health, which highlight how socioeconomic inequities contribute to disparities in obesity and related metabolic conditions [31]. The observed associations may be attributed to differences in nutritional status and access to healthcare among rural and low‐education populations [32, 33]. Given these findings, targeted health promotion strategies—such as high‐nutrient dietary interventions, vitamin D supplementation and resistance training programs—should be prioritized for socioeconomically disadvantaged groups at high risk of low appendicular circumferences.

Although higher BMI was inversely associated with low appendicular circumferences (CC, TC, UAC, FC), elevated body fat percentage showed a positive correlation specifically with low CC. This discrepancy was particularly pronounced in individuals aged ≥ 60 years, suggesting that BMI‐based sarcopenia risk stratification in older adults requires further compositional refinement (e.g., distinguishing lean vs. fat mass contributions). Our findings align with established evidence linking metabolic disorders to sarcopenia pathogenesis [2], where chronic inflammation, oxidative stress and endothelial dysfunction are implicated [34, 35]. Specifically, DM was significantly associated with low TC, CC and UAC in our study—consistent with reports linking reduced TC to DM [36, 37] and demonstrating that diminished femoral subcutaneous adipose tissue correlates with dysglycemia/dyslipidemia [38]. Mechanistically, lower muscle mass exacerbates insulin resistance [39] and potentiates cardiometabolic dysfunction [5, 37], highlighting bidirectional muscle–metabolism interactions.

Building on these findings, future research should prioritize validating our age‐stratified cutoffs within longitudinal cohorts to establish diagnostic thresholds for Chinese‐specific sarcopenia criteria. Additionally, mechanistic studies exploring biological drivers of site‐specific muscle loss patterns, for example, differential fat infiltration in thigh vs. calf, could inform targeted interventions.

This study establishes the first nationally representative, age‐ and sex‐stratified reference percentiles for key appendicular circumferences (thigh, calf, upper arm, forearm) in Chinese adults. These normative data provide essential benchmarks for clinical risk stratification, public health surveillance and lifestyle intervention evaluation. The identified risk associations further enable precision screening strategies for sarcopenia prevention. Limitations include the use of BIA rather than DXA for body composition assessment, potentially introducing variability in muscle/fat quantification; however, BIA's practicality for large‐scale screening aligns with our study objectives and is unlikely to compromise primary circumference references. Secondly, the cross‐sectional design precludes causal inference—future longitudinal studies should validate predictive relationships between identified risk factors and incident muscle loss.

## 5 Conclusions

This study established comprehensive sex‐ and age‐specific normative values for TC, CC, UAC and FC in Chinese adults. Our findings demonstrated significant sex differences in both absolute and relative appendicular circumferences. These normative data serve as valuable reference standards for evaluating upper and lower appendicular circumferences in general adult populations. Importantly, when integrated with additional parameters such as body composition analysis and handgrip strength measurements, these circumference references may significantly enhance early identification of high‐risk individuals for sarcopenia, thereby facilitating timely preventive interventions.

## Conflicts of Interest

The authors declare no conflicts of interest.

## Supporting information


**Table S1:** Basic characteristics of the reference population.
**Table S2:** Basic characteristics of the association study population.
**Table S3:** Factors associated with low appendicular circumferences in people aged over 60 years.
**Table S4:** Factors associated with low appendicular circumferences in people aged 41~60 years.
**Table S5:** Factors associated with low appendicular circumferences in people aged ≤ 40 years.
**Table S6:** Sensitivity analysis of factors associated with low appendicular circumferences in participants without cardiometabolic disorders, cancer or musculoskeletal diseases.
**Figure S1:** Comparison of appendicular circumferences between age‐matched males and females.
**Figure S2:** Comparison of appendicular circumferences, muscle mass and strength between age‐matched postmenopausal and premenopausal women. Menopause status: 0 = premenopause, 1 = postmenopause. ASM: appendicular skeletal muscle mass; ASMI: appendicular skeletal muscle mass index; %BF: body fat percentage; CC: calf circumference; FC: forearm circumference; TC: thigh circumference; UAC: upper arm circumference.
