# Supplementary material for: 3D Body Scanning–Derived Normative Values of Appendicular Circumferences: A Novel Tool for Sarcopenia Screening in Chinese Adults
Source: J Cachexia Sarcopenia Muscle. 2025 Nov 16;16(6):e70135. doi: 10.1002/jcsm.70135 (PMC12620412; doi:10.1002/jcsm.70135)
Supplement: Supplementary file 1 — Table S1: Basic characteristics of the reference population. Table S2: Basic characteristics of the association study population. Table S3: Factors associated with low appendicular circumferences in people aged over 60 years. Table S4: Factors associated with low appendicular circumferences in people aged 41~60 years. Table S5: Factors associated with low appendicular circumferences in people aged ≤ 40 years. Table S6: Sensitivity analysis of factors associated with low appendicular circumferences in participants without cardiometabolic disorders, cancer or musculoskeletal diseases. Figure S1: Comparison of appendicular circumferences between age‐matched males and females. Figure S2: Comparison of appendicular circumferences, muscle mass and strength between age‐matched postmenopausal and premenopausal women. Menopause status: 0 = premenopause, 1 = postmenopause. ASM: appendicular skeletal muscle mass; ASMI: appendicular skeletal muscle mass index; %BF: body fat percentage; CC: calf circumference; FC: forearm circumference; TC: thigh circumference; UAC: upper arm circumference. [file JCSM-16-e70135-s001.docx]

**Supplementary materials**

**Table S1** Basic characteristics of the reference population.

| Variables | Male (n=3510) | | Female (n=5405) | | Overall (n=8915) | |
| --- | --- | --- | --- | --- | --- | --- |
| **Age** | 53.8 | 13.19 | 53.2 | 12.43 | 53.44 | 12.74 |
| 20- | 184 | 5.24 | 215 | 3.98 | 399 | 4.48 |
| 30- | 435 | 12.39 | 697 | 12.90 | 1132 | 12.70 |
| 40- | 625 | 17.81 | 1067 | 19.74 | 1692 | 18.98 |
| 50- | 990 | 28.21 | 1655 | 30.62 | 2645 | 29.67 |
| 60- | 941 | 26.81 | 1341 | 24.81 | 2282 | 25.60 |
| 70- | 335 | 9.54 | 430 | 7.96 | 765 | 8.58 |
| **Urban** | 2063 | 58.77 | 3504 | 64.83 | 5567 | 62.45 |
| **Rural** | 1424 | 40.57 | 1862 | 34.45 | 3286 | 36.86 |
| **Education** |  |  |  |  |  |  |
| Illiterate | 475 | 13.53 | 849 | 15.71 | 1324 | 14.85 |
| Elementary school | 1168 | 33.28 | 1641 | 30.36 | 2809 | 31.51 |
| High school | 790 | 22.51 | 1270 | 23.50 | 2060 | 23.11 |
| College or above | 1068 | 30.43 | 1630 | 30.16 | 2698 | 30.26 |
| **Health-related lifestyles** |  |  |  |  |  |  |
| **Cigarette smoking** |  |  |  |  |  |  |
| Never smoker | 1290 | 36.75 | 5216 | 96.50 | 6506 | 72.98 |
| Former smoker | 600 | 17.09 | 40 | 0.74 | 640 | 7.18 |
| Current smoker | 1619 | 46.13 | 147 | 2.72 | 1766 | 19.81 |
| **Alcohol drink** |  |  |  |  |  |  |
| Never drinker | 1212 | 34.53 | 4856 | 89.84 | 6068 | 68.07 |
| Former drinker | 275 | 7.83 | 50 | 0.93 | 325 | 3.65 |
| Current drinker | 2021 | 57.58 | 497 | 9.20 | 2518 | 28.24 |
| **Physical exercise** |  |  |  |  |  |  |
| 5~7 days per week | 1501 | 42.76 | 2369 | 43.83 | 3870 | 43.41 |
| 3~4 days per week | 340 | 9.69 | 545 | 10.08 | 885 | 9.93 |
| 1~2 days per week | 404 | 11.51 | 575 | 10.64 | 979 | 10.98 |
| ≤ 3 days per month | 254 | 7.24 | 394 | 7.29 | 648 | 7.27 |
| Never | 1008 | 28.72 | 1520 | 28.12 | 2528 | 28.36 |
| **Anthropometry measurement** | |  |  |  |  |  |
| Height | 169.28 | 6.71 | 157.19 | 6.06 | 161.95 | 8.66 |
| Weight | 72.83 | 12.53 | 60.39 | 9.66 | 65.29 | 12.46 |
| Chest circumference | 99.90 | 8.63 | 94.30 | 8.55 | 96.52 | 9.00 |
| Waist circumference | 87.84 | 9.50 | 80.65 | 8.77 | 83.49 | 9.73 |
| Hip circumference | 97.76 | 6.49 | 96.94 | 6.86 | 97.27 | 6.73 |
| Abdominal circumference | 92.6 | 9.26 | 90.46 | 8.78 | 91.31 | 9.03 |
| Left upper arm circumference | 27.93 | 3.88 | 27.94 | 6.54 | 27.93 | 5.65 |
| Right upper arm circumference | 28.29 | 4.01 | 27.64 | 6.18 | 27.90 | 5.44 |
| Left forearm circumference | 26.42 | 3.98 | 24.65 | 6.60 | 25.35 | 5.78 |
| Right forearm circumference | 26.88 | 3.45 | 25.00 | 6.53 | 25.74 | 5.60 |
| Left thigh circumference | 53.82 | 6.24 | 52.12 | 4.98 | 52.79 | 5.58 |
| Right thigh circumference | 54.44 | 6.24 | 52.50 | 5.07 | 53.27 | 5.64 |
| Left calf circumference | 36.90 | 3.87 | 35.60 | 3.62 | 36.11 | 3.77 |
| Right calf circumference | 36.96 | 3.57 | 35.66 | 3.27 | 36.17 | 3.46 |
| **Body composition** |  |  |  |  |  |  |
| Body fat percentage (%) | 22.40 | 5.92 | 33.38 | 6.45 | 29.07 | 8.23 |
| Fat mass (kg) | 16.91 | 6.94 | 20.68 | 7.04 | 19.20 | 7.24 |
| Fat free mass (kg) | 55.94 | 6.56 | 39.72 | 3.62 | 46.09 | 9.36 |
| Muscle mass (kg) | 53.03 | 6.23 | 37.4 | 3.31 | 43.54 | 8.95 |
| Body mass index (kg/m^2^) | 25.34 | 3.63 | 24.43 | 3.57 | 24.79 | 3.62 |
| Right leg fat percentage (%) | 22.06 | 4.64 | 34.85 | 4.22 | 29.82 | 7.64 |
| Left leg fat percentage (%) | 21.94 | 4.69 | 34.87 | 4.19 | 29.79 | 7.69 |
| Right leg fat mass (kg) | 2.99 | 1.19 | 3.78 | 0.99 | 3.47 | 1.14 |
| Left leg fat mass (kg) | 2.97 | 1.16 | 3.73 | 0.97 | 3.43 | 1.12 |
| Right leg fat free mass (kg) | 10.21 | 1.67 | 6.93 | 0.86 | 8.22 | 2.03 |
| Left leg fat free mass (kg) | 10.08 | 1.68 | 6.85 | 0.87 | 8.11 | 2.01 |
| Right leg muscle mass (kg) | 9.65 | 1.58 | 6.53 | 0.80 | 7.75 | 1.92 |
| Right arm fat percentage (%) | 9.52 | 1.59 | 6.45 | 0.80 | 7.66 | 1.91 |
| Left arm fat percentage (%) | 16.47 | 4.60 | 29.91 | 7.44 | 24.63 | 9.22 |
| Right arm fat mass (kg) | 17.65 | 4.95 | 31.1 | 7.51 | 25.82 | 9.33 |
| Left arm fat mass (kg) | 0.63 | 0.26 | 0.91 | 0.45 | 0.80 | 0.41 |
| Right arm fat free mass (kg) | 0.66 | 0.28 | 0.93 | 0.45 | 0.83 | 0.42 |
| Left arm fat free mass (kg) | 3.05 | 0.42 | 1.99 | 0.29 | 2.41 | 0.62 |
| Right arm muscle mass (kg) | 2.95 | 0.40 | 1.92 | 0.28 | 2.33 | 0.60 |
| Left arm muscle mass (kg) | 2.85 | 0.41 | 1.87 | 0.26 | 2.26 | 0.58 |
| **Handgrip Strength** | 43.18 | 8.89 | 26.96 | 5.38 | 33.36 | 10.56 |
| **Clinical profiles** |  |  |  |  |  |  |
| SBP | 131.07 | 16.69 | 123.92 | 18.74 | 126.74 | 18.30 |
| DBP | 81.12 | 11.00 | 75.79 | 10.64 | 77.89 | 11.09 |
| TC | 5.20 | 1.07 | 5.45 | 1.07 | 5.35 | 1.08 |
| TG | 2.03 | 1.75 | 1.63 | 1.13 | 1.79 | 1.42 |
| LDL-C | 2.95 | 0.83 | 3.15 | 0.87 | 3.07 | 0.86 |
| HDL-C | 1.22 | 0.32 | 1.41 | 0.35 | 1.34 | 0.35 |
| FPG | 5.88 | 1.73 | 5.58 | 1.22 | 5.70 | 1.45 |
| UA | 397.37 | 91.58 | 310.55 | 73.23 | 344.74 | 91.39 |
| **Disease history** |  |  |  |  |  |  |
| Obesity | 734 | 20.91 | 827 | 15.30 | 1561 | 17.51 |
| HTN | 1588 | 45.24 | 1773 | 32.80 | 3361 | 37.70 |
| DM | 521 | 14.84 | 553 | 10.23 | 1074 | 12.05 |
| Dyslipidemia | 1627 | 46.35 | 1989 | 36.80 | 3616 | 40.56 |
| HUA | 1308 | 37.26 | 1232 | 22.79 | 2540 | 28.49 |

SBP: systolic blood pressure; DBP: diastolic blood pressure; TC: total cholesterol; TG: triglycerides; LDL-C: low-density lipoprotein cholesterol; HDL-C: high-density lipoprotein cholesterol; FPG: fasting plasma glucose; UA: uric acid; HTN: hypertension; DM: diabetes mellitus; HUA: hyperuricemia.

**Table S2** Basic characteristics of the association study population.

| Variables | Male (n = 4378) | | Female (n = 6254) | | Overall (n = 10632) | |
| --- | --- | --- | --- | --- | --- | --- |
| **Age** | 54.98 | 13.13 | 54.62 | 12.54 | 54.77 | 12.79 |
| 20- | 197 | 4.50 | 215 | 3.44 | 412 | 3.88 |
| 30- | 497 | 11.35 | 710 | 11.35 | 1207 | 11.35 |
| 40- | 710 | 16.22 | 1120 | 17.91 | 1830 | 17.21 |
| 50- | 1202 | 27.46 | 1852 | 29.61 | 3054 | 28.72 |
| 60- | 1273 | 29.08 | 1707 | 27.29 | 2980 | 28.03 |
| 70- | 499 | 11.40 | 650 | 10.39 | 1149 | 10.81 |
| **Urban** | 2580 | 58.93 | 4040 | 64.60 | 6620 | 62.26 |
| **Rural** | 1768 | 40.38 | 2172 | 34.73 | 3940 | 37.06 |
| **Education** |  |  |  |  |  |  |
| Illiterate | 604 | 13.80 | 1061 | 16.97 | 1665 | 15.66 |
| Elementary school | 1476 | 33.71 | 1918 | 30.67 | 3394 | 31.92 |
| High school | 1007 | 23.00 | 1512 | 24.18 | 2519 | 23.69 |
| College or above | 1280 | 29.24 | 1746 | 27.92 | 3026 | 28.46 |
| **Health-related lifestyles** |  |  |  |  |  |  |
| **Cigarette smoking** |  |  |  |  |  |  |
| Never smoke | 1550 | 35.40 | 6009 | 96.08 | 7559 | 71.10 |
| Quit smoke | 868 | 19.83 | 55 | 0.88 | 923 | 8.68 |
| Current smoke | 1959 | 44.75 | 188 | 3.01 | 2147 | 20.19 |
| **Alcohol drink** |  |  |  |  |  |  |
| Never drink | 1445 | 33.01 | 5635 | 90.10 | 7080 | 66.59 |
| Quit drink | 433 | 9.89 | 73 | 1.17 | 506 | 4.76 |
| Current drink | 2497 | 57.04 | 544 | 8.70 | 3041 | 28.60 |
| **Physical exercise** |  |  |  |  |  |  |
| 5~7 days per week | 1983 | 45.29 | 2825 | 45.17 | 4808 | 45.22 |
| 3~4 days per week | 392 | 8.95 | 594 | 9.50 | 986 | 9.27 |
| 1~2 days per week | 473 | 10.80 | 639 | 10.22 | 1112 | 10.46 |
| ≤ 3 days per month | 302 | 6.90 | 450 | 7.20 | 752 | 7.07 |
| Never | 1224 | 27.96 | 1744 | 27.89 | 2968 | 27.92 |
| **Anthropometry measurement** | |  |  |  |  |  |
| Height | 169.09 | 6.71 | 157.02 | 6.07 | 161.99 | 8.69 |
| Weight | 72.96 | 12.55 | 60.51 | 9.66 | 65.63 | 12.54 |
| Chest circumference | 100.12 | 8.68 | 94.54 | 8.52 | 96.85 | 9.02 |
| Waist circumference | 88.22 | 9.49 | 81.14 | 8.83 | 84.07 | 9.75 |
| Hip circumference | 97.88 | 6.56 | 97.08 | 6.93 | 97.41 | 6.79 |
| Abdominal circumference | 92.95 | 9.31 | 90.90 | 8.79 | 91.75 | 9.07 |
| Left upper arm circumference | 27.93 | 3.73 | 28.00 | 6.79 | 27.97 | 5.73 |
| Right upper arm circumference | 28.26 | 3.80 | 27.69 | 6.30 | 27.92 | 5.42 |
| Left forearm circumference | 26.38 | 3.69 | 24.69 | 6.88 | 25.39 | 5.85 |
| Right forearm circumference | 26.82 | 3.26 | 25.00 | 6.61 | 25.75 | 5.56 |
| Left thigh circumference | 53.71 | 6.32 | 51.97 | 5.01 | 52.69 | 5.65 |
| Right thigh circumference | 54.33 | 6.21 | 52.36 | 5.09 | 53.17 | 5.66 |
| Left calf circumference | 36.87 | 3.91 | 35.53 | 3.63 | 36.09 | 3.81 |
| Right calf circumference | 36.94 | 3.60 | 35.59 | 3.26 | 36.15 | 3.47 |
| **Body composition** |  |  |  |  |  |  |
| Body fat percentage (%) | 22.66 | 5.92 | 33.60 | 6.49 | 29.10 | 8.26 |
| Fat mass (kg) | 17.11 | 6.98 | 20.86 | 7.07 | 19.32 | 7.27 |
| Fat free mass (kg) | 55.85 | 6.57 | 39.66 | 3.63 | 46.31 | 9.43 |
| Muscle mass (kg) | 52.95 | 6.25 | 37.35 | 3.32 | 43.76 | 9.02 |
| Body mass index (kg/m^2^) | 25.44 | 3.65 | 24.53 | 3.57 | 24.90 | 3.63 |
| Right leg fat percentage (%) | 22.24 | 4.63 | 35.01 | 4.26 | 29.76 | 7.68 |
| Left leg fat percentage (%) | 22.11 | 4.68 | 35.03 | 4.23 | 29.72 | 7.74 |
| Right leg fat mass (kg) | 3.02 | 1.20 | 3.79 | 0.99 | 3.47 | 1.14 |
| Left leg fat mass (kg) | 2.99 | 1.17 | 3.74 | 0.98 | 3.43 | 1.12 |
| Right leg fat free mass (kg) | 10.19 | 1.69 | 6.89 | 0.87 | 8.25 | 2.06 |
| Left leg fat free mass (kg) | 10.05 | 1.69 | 6.81 | 0.87 | 8.14 | 2.04 |
| Right leg muscle mass (kg) | 9.63 | 1.60 | 6.49 | 0.80 | 7.78 | 1.95 |
| Right arm fat percentage (%) | 9.50 | 1.60 | 6.41 | 0.81 | 7.68 | 1.93 |
| Left arm fat percentage (%) | 16.71 | 4.61 | 30.19 | 7.47 | 24.65 | 9.25 |
| Right arm fat mass (kg) | 17.91 | 4.97 | 31.39 | 7.57 | 25.85 | 9.37 |
| Left arm fat mass (kg) | 0.64 | 0.26 | 0.93 | 0.45 | 0.81 | 0.41 |
| Right arm fat free mass (kg) | 0.67 | 0.28 | 0.95 | 0.46 | 0.83 | 0.42 |
| Left arm fat free mass (kg) | 3.05 | 0.42 | 1.99 | 0.29 | 2.43 | 0.63 |
| Right arm muscle mass (kg) | 2.95 | 0.40 | 1.93 | 0.29 | 2.35 | 0.61 |
| Left arm muscle mass (kg) | 2.85 | 0.41 | 1.88 | 0.26 | 2.27 | 0.58 |
| **Handgrip Strength** | 42.71 | 8.94 | 26.59 | 5.45 | 33.23 | 10.64 |
| **Clinical profiles** | 131.76 | 17.08 | 124.85 | 18.94 | 127.70 | 18.51 |
| SBP | 81.23 | 11.18 | 75.96 | 10.66 | 78.13 | 11.18 |
| DBP | 5.15 | 1.12 | 5.46 | 1.09 | 5.33 | 1.11 |
| TC | 2.03 | 1.74 | 1.66 | 1.14 | 1.81 | 1.43 |
| TG | 2.91 | 0.87 | 3.15 | 0.88 | 3.05 | 0.88 |
| LDL | 1.22 | 0.33 | 1.41 | 0.35 | 1.33 | 0.35 |
| HDL | 5.91 | 1.72 | 5.62 | 1.25 | 5.74 | 1.47 |
| FPG | 42.71 | 8.94 | 26.59 | 5.45 | 33.23 | 10.64 |
| UA | 403.39 | 97.74 | 312.28 | 74.56 | 349.81 | 95.99 |
| **Disease history** |  |  |  |  |  |  |
| Obesity | 945 | 21.59 | 999 | 15.97 | 1944 | 18.28 |
| HTN | 2162 | 49.38 | 2262 | 36.17 | 4424 | 41.61 |
| DM | 723 | 16.51 | 745 | 11.91 | 1468 | 13.81 |
| Dyslipidemia | 2048 | 46.78 | 2374 | 37.96 | 4422 | 41.59 |
| HUA | 1714 | 39.15 | 1481 | 23.68 | 3195 | 30.05 |

SBP: systolic blood pressure; DBP: diastolic blood pressure; TC: total cholesterol; TG: triglycerides; LDL-C: low-density lipoprotein cholesterol; HDL-C: high-density lipoprotein cholesterol; FPG: fasting plasma glucose; UA: uric acid; HTN: hypertension; DM: diabetes mellitus; HUA: hyperuricemia.

**Table S3** Factors associated with low appendicular circumferences in people aged over 60 years.

|  | **Low calf circumference** | | | | **Low thigh circumference** | | | | **Low upper arm circumference** | | | | **Low forearm circumference** | | | |
| --- | --- | --- | --- | --- | --- | --- | --- | --- | --- | --- | --- | --- | --- | --- | --- | --- |
|  | OR | 95% CI | | *p* | OR | 95% CI | | *p* | OR | 95% CI | | *p* | OR | 95% CI | | *p* |
| **Urban** | Ref | NA | | NA | Ref | NA | | NA | Ref | NA | | NA | Ref | NA | | NA |
| **Rural** | 2.540 | 1.601 | 4.028 | <0.001 | 1.814 | 1.112 | 2.961 | 0.017 | 0.642 | 0.386 | 1.068 | 0.088 | 0.828 | 0.520 | 1.318 | 0.426 |
| **Education** |  |  |  |  |  |  |  |  |  |  |  |  |  |  |  |  |
| Illiterate | 0.673 | 0.324 | 1.397 | 0.288 | 0.877 | 0.403 | 1.909 | 0.741 | 0.668 | 0.319 | 1.401 | 0.286 | 0.450 | 0.223 | 0.905 | 0.025 |
| Elementary school | 0.907 | 0.479 | 1.718 | 0.765 | 0.898 | 0.455 | 1.772 | 0.756 | 0.784 | 0.424 | 1.449 | 0.437 | 0.625 | 0.352 | 1.110 | 0.109 |
| High school | 0.647 | 0.339 | 1.235 | 0.187 | 0.846 | 0.433 | 1.653 | 0.625 | 0.638 | 0.348 | 1.170 | 0.147 | 0.646 | 0.369 | 1.130 | 0.126 |
| College or above | Ref | NA | | NA | Ref | NA | | NA | Ref | NA | | NA | Ref | NA | | NA |
| BMI | 0.411 | 0.348 | 0.486 | <0.001 | 0.483 | 0.404 | 0.576 | <0.001 | 0.519 | 0.436 | 0.618 | <0.001 | 0.714 | 0.613 | 0.832 | <0.001 |
| %BF | 1.115 | 1.060 | 1.172 | <0.001 | 0.960 | 0.909 | 1.012 | 0.132 | 0.958 | 0.908 | 1.010 | 0.112 | 0.933 | 0.887 | 0.982 | 0.008 |
| Muscle mass | 0.973 | 0.922 | 1.026 | 0.311 | 0.888 | 0.838 | 0.942 | <0.001 | 0.851 | 0.801 | 0.903 | <0.001 | 0.837 | 0.792 | 0.885 | <0.001 |
| **Smoking status** |  |  |  |  |  |  |  |  |  |  |  |  |  |  |  |  |
| Never | Ref | NA | | NA | Ref | NA | | Ref | Ref | NA | | NA | Ref | NA | | NA |
| Former | 2.911 | 1.449 | 5.845 | 0.003 | 1.233 | 0.571 | 2.664 | 0.594 | 2.361 | 1.096 | 5.083 | 0.028 | 3.909 | 1.962 | 7.790 | <0.001 |
| Current | 2.624 | 1.488 | 4.629 | 0.001 | 1.175 | 0.643 | 2.146 | 0.601 | 2.525 | 1.363 | 4.677 | 0.003 | 3.082 | 1.723 | 5.513 | <0.001 |
| **Alcohol drinking** |  |  |  |  |  |  |  |  |  |  |  |  |  |  |  |  |
| Never | Ref | NA | | NA | Ref | NA | | Ref | Ref | NA | | NA | Ref | NA | | NA |
| Former | 0.661 | 0.288 | 1.519 | 0.330 | 0.881 | 0.376 | 2.066 | 0.771 | 1.028 | 0.436 | 2.424 | 0.951 | 0.531 | 0.232 | 1.215 | 0.134 |
| Current | 0.991 | 0.579 | 1.694 | 0.973 | 1.270 | 0.729 | 2.213 | 0.399 | 1.828 | 1.061 | 3.148 | 0.030 | 0.753 | 0.443 | 1.279 | 0.294 |
| **Physical exercise** |  |  |  |  |  |  |  |  |  |  |  |  |  |  |  |  |
| 5~7 days per week | Ref | NA | | NA | Ref | NA | | Ref | Ref | NA | | NA | Ref | NA | | NA |
| 3~4 days per week | 0.869 | 0.355 | 2.126 | 0.758 | 0.470 | 0.157 | 1.413 | 0.179 | 1.532 | 0.718 | 3.271 | 0.270 | 0.698 | 0.300 | 1.625 | 0.404 |
| 1~2 days per week | 0.944 | 0.409 | 2.182 | 0.894 | 1.157 | 0.497 | 2.693 | 0.736 | 0.924 | 0.379 | 2.253 | 0.862 | 0.787 | 0.346 | 1.786 | 0.566 |
| ≤ 3 days per month | 1.110 | 0.425 | 2.902 | 0.831 | 1.737 | 0.648 | 4.657 | 0.272 | 0.784 | 0.249 | 2.471 | 0.677 | 0.656 | 0.222 | 1.935 | 0.445 |
| Never | 1.583 | 1.039 | 2.411 | 0.033 | 1.351 | 0.852 | 2.142 | 0.201 | 1.118 | 0.691 | 1.810 | 0.650 | 1.140 | 0.736 | 1.766 | 0.558 |
| HTN | 1.453 | 0.997 | 2.117 | 0.052 | 1.686 | 1.128 | 2.520 | 0.011 | 1.261 | 0.849 | 1.873 | 0.251 | 1.244 | 0.866 | 1.787 | 0.238 |
| DM | 2.266 | 1.453 | 3.535 | <0.001 | 4.821 | 3.054 | 7.609 | <0.001 | 1.377 | 0.829 | 2.288 | 0.217 | 0.699 | 0.411 | 1.189 | 0.186 |
| HUA | 1.362 | 0.850 | 2.184 | 0.199 | 1.302 | 0.775 | 2.188 | 0.318 | 1.169 | 0.692 | 1.975 | 0.560 | 0.791 | 0.476 | 1.315 | 0.366 |
| Low HDL | 0.951 | 0.483 | 1.872 | 0.884 | 2.377 | 1.283 | 4.406 | 0.006 | 1.163 | 0.577 | 2.345 | 0.674 | 1.261 | 0.675 | 2.356 | 0.468 |
| High non-HDL-C | 1.029 | 0.652 | 1.625 | 0.903 | 1.117 | 0.685 | 1.821 | 0.659 | 0.739 | 0.439 | 1.242 | 0.254 | 0.827 | 0.521 | 1.312 | 0.419 |

HTN: hypertension; DM: diabetes mellitus; HUA: hyperuricemia; HDL-C: high density lipoprotein cholesterol; OR: odds ratio; CI: confidence interval.

**Table S4** Factors associated with low appendicular circumferences in people aged 41~60 years.

|  | **Low calf circumference** | | | | **Low thigh circumference** | | | | **Low upper arm circumference** | | | | **Low forearm circumference** | | | |
| --- | --- | --- | --- | --- | --- | --- | --- | --- | --- | --- | --- | --- | --- | --- | --- | --- |
|  | OR | 95% CI | | *p* | OR | 95% CI | | *p* | OR | 95% CI | | *p* | OR | 95% CI | | *p* |
| **Urban** | Ref | NA | | NA | Ref | NA | | NA | Ref | NA | | NA | Ref | NA | | NA |
| **Rural** | 1.491 | 1.017 | 2.187 | 0.041 | 1.202 | 0.786 | 1.839 | 0.397 | 0.768 | 0.502 | 1.176 | 0.225 | 0.707 | 0.466 | 1.073 | 0.103 |
| **Education** |  |  |  |  |  |  |  |  |  |  |  |  |  |  |  |  |
| Illiterate | 1.863 | 1.059 | 3.279 | 0.031 | 0.974 | 0.511 | 1.858 | 0.936 | 0.664 | 0.347 | 1.273 | 0.218 | 0.590 | 0.309 | 1.128 | 0.111 |
| Elementary school | 1.335 | 0.860 | 2.073 | 0.197 | 1.123 | 0.698 | 1.807 | 0.633 | 0.741 | 0.474 | 1.159 | 0.189 | 0.841 | 0.543 | 1.301 | 0.436 |
| High school | 0.864 | 0.547 | 1.366 | 0.531 | 0.876 | 0.546 | 1.407 | 0.585 | 0.770 | 0.503 | 1.177 | 0.228 | 0.869 | 0.572 | 1.320 | 0.510 |
| College or above | Ref | NA | | NA | Ref | NA | | NA | Ref | NA | | NA | Ref | NA | | NA |
| BMI | 0.458 | 0.395 | 0.531 | <0.001 | 0.443 | 0.374 | 0.525 | <0.001 | 0.548 | 0.470 | 0.639 | <0.001 | 0.559 | 0.481 | 0.650 | <0.001 |
| %BF | 1.054 | 1.004 | 1.106 | 0.034 | 0.940 | 0.892 | 0.991 | 0.022 | 0.924 | 0.878 | 0.972 | 0.002 | 0.947 | 0.901 | 0.997 | 0.037 |
| Muscle mass | 0.967 | 0.921 | 1.014 | 0.167 | 0.880 | 0.833 | 0.930 | <0.001 | 0.900 | 0.854 | 0.948 | <0.001 | 0.859 | 0.814 | 0.906 | <0.001 |
| **Ever smoke** | 2.403 | 1.443 | 4.003 | 0.001 | 3.285 | 1.873 | 5.761 | <0.001 | 1.654 | 0.968 | 2.829 | 0.066 | 4.047 | 2.299 | 7.125 | <0.001 |
| **Ever drink** | 1.319 | 0.845 | 2.058 | 0.223 | 1.111 | 0.686 | 1.799 | 0.668 | 1.559 | 0.997 | 2.436 | 0.051 | 1.093 | 0.690 | 1.731 | 0.705 |
| **Physical exercise** |  |  |  |  |  |  |  |  |  |  |  |  |  |  |  |  |
| 5~7 days per week | Ref | NA | | NA | Ref | NA | | Ref | Ref | NA | | NA | Ref | NA | | NA |
| 3~4 days per week | 0.670 | 0.366 | 1.226 | 0.194 | 0.916 | 0.503 | 1.666 | 0.773 | 0.722 | 0.411 | 1.267 | 0.256 | 0.497 | 0.271 | 0.913 | 0.024 |
| 1~2 days per week | 0.685 | 0.372 | 1.263 | 0.226 | 1.075 | 0.595 | 1.942 | 0.811 | 0.521 | 0.280 | 0.971 | 0.040 | 0.675 | 0.379 | 1.203 | 0.183 |
| ≤ 3 days per month | 0.935 | 0.515 | 1.700 | 0.827 | 0.467 | 0.215 | 1.014 | 0.054 | 1.225 | 0.687 | 2.182 | 0.492 | 0.681 | 0.358 | 1.296 | 0.242 |
| Never | 1.131 | 0.783 | 1.635 | 0.511 | 1.012 | 0.670 | 1.529 | 0.954 | 0.674 | 0.446 | 1.017 | 0.060 | 0.791 | 0.536 | 1.167 | 0.237 |
| HTN | 1.486 | 1.013 | 2.179 | 0.043 | 2.031 | 1.329 | 3.104 | 0.001 | 0.686 | 0.431 | 1.092 | 0.112 | 1.472 | 0.983 | 2.206 | 0.061 |
| DM | 1.837 | 1.087 | 3.103 | 0.023 | 4.401 | 2.599 | 7.453 | <0.001 | 2.956 | 1.718 | 5.088 | <0.001 | 1.354 | 0.743 | 2.465 | 0.322 |
| HUA | 1.339 | 0.890 | 2.015 | 0.161 | 1.425 | 0.899 | 2.257 | 0.132 | 1.436 | 0.925 | 2.229 | 0.107 | 1.509 | 0.986 | 2.310 | 0.058 |
| Low HDL | 1.059 | 0.586 | 1.916 | 0.849 | 1.373 | 0.720 | 2.620 | 0.336 | 0.620 | 0.297 | 1.295 | 0.203 | 0.881 | 0.449 | 1.729 | 0.714 |
| High non-HDL-C | 1.497 | 1.005 | 2.228 | 0.047 | 1.266 | 0.787 | 2.037 | 0.332 | 1.241 | 0.790 | 1.949 | 0.348 | 1.068 | 0.683 | 1.671 | 0.774 |

HTN: hypertension; DM: diabetes mellitus; HUA: hyperuricemia; HDL-C: high density lipoprotein cholesterol; OR: odds ratio; CI: confidence interval.

**Table S5** Factors associated with low appendicular circumferences in people aged ≤ 40 years.

|  | **Low calf circumference** | | | | **Low thigh circumference** | | | | **Low upper arm circumference** | | | | **Low forearm circumference** | | | |
| --- | --- | --- | --- | --- | --- | --- | --- | --- | --- | --- | --- | --- | --- | --- | --- | --- |
|  | OR | 95% CI | | *p* | OR | 95% CI | | *p* | OR | 95% CI | | *p* | OR | 95% CI | | *p* |
| **Urban** | Ref | NA | | NA | Ref | NA | | NA | Ref | NA | | NA | Ref | NA | | NA |
| **Rural** | 0.952 | 0.482 | 1.880 | 0.887 | 0.878 | 0.398 | 1.935 | 0.747 | 0.279 | 0.123 | 0.637 | 0.002 | 0.270 | 0.117 | 0.625 | 0.002 |
| **Education** |  |  |  |  |  |  |  |  |  |  |  |  |  |  |  |  |
| High school or lower | 1.488 | 0.747 | 2.965 | 0.258 | 1.666 | 0.743 | 3.736 | 0.215 | 1.134 | 0.531 | 2.422 | 0.746 | 1.271 | 0.750 | 2.152 | 0.373 |
| College or above | Ref | NA | | NA | Ref | NA | | NA | Ref | NA | | NA | Ref | NA | | NA |
| BMI | 0.431 | 0.323 | 0.577 | <0.001 | 0.396 | 0.272 | 0.575 | <0.001 | 0.511 | 0.376 | 0.695 | <0.001 | 0.669 | 0.502 | 0.893 | 0.006 |
| %BF | 1.031 | 0.945 | 1.123 | 0.494 | 0.871 | 0.783 | 0.969 | 0.011 | 0.896 | 0.817 | 0.984 | 0.021 | 0.848 | 0.772 | 0.930 | 0.001 |
| Muscle mass | 1.007 | 0.924 | 1.096 | 0.881 | 0.908 | 0.815 | 1.012 | 0.082 | 0.929 | 0.846 | 1.021 | 0.126 | 0.868 | 0.789 | 0.955 | 0.004 |
| **Ever smoke** | 2.156 | 0.858 | 5.416 | 0.102 | 2.202 | 0.733 | 6.616 | 0.160 | 1.553 | 0.584 | 4.128 | 0.377 | 1.205 | 0.462 | 3.142 | 0.703 |
| **Ever drink** | 2.153 | 1.086 | 4.270 | 0.028 | 1.310 | 0.555 | 3.095 | 0.538 | 0.778 | 0.353 | 1.710 | 0.532 | 1.233 | 0.597 | 2.548 | 0.571 |
| **Physical exercise** |  |  |  |  |  |  |  |  |  |  |  |  |  |  |  |  |
| 5~7 days per week | Ref | NA | | NA | Ref | NA | | Ref | Ref | NA | | NA | Ref | NA | | NA |
| 3~4 days per week | 1.645 | 0.642 | 4.213 | 0.300 | 1.139 | 0.347 | 3.740 | 0.830 | 1.289 | 0.442 | 3.760 | 0.642 | 0.946 | 0.347 | 2.576 | 0.913 |
| 1~2 days per week | 1.308 | 0.526 | 3.253 | 0.564 | 0.757 | 0.245 | 2.341 | 0.629 | 2.195 | 0.830 | 5.801 | 0.113 | 1.506 | 0.612 | 3.709 | 0.373 |
| ≤ 3 days per month | 1.007 | 0.379 | 2.679 | 0.988 | 1.105 | 0.381 | 3.207 | 0.854 | 1.091 | 0.373 | 3.194 | 0.873 | 0.662 | 0.230 | 1.909 | 0.446 |
| Never | 1.046 | 0.477 | 2.293 | 0.910 | 0.881 | 0.359 | 2.163 | 0.782 | 1.551 | 0.667 | 3.604 | 0.308 | 1.055 | 0.479 | 2.324 | 0.894 |
| HUA | 0.675 | 0.299 | 1.523 | 0.344 | 2.052 | 0.822 | 5.118 | 0.123 | 1.825 | 0.805 | 4.141 | 0.150 | 2.852 | 1.333 | 6.102 | 0.007 |

HUA: hyperuricemia; OR: odds ratio; CI: confidence interval. Diabetes and dyslipidemia were not set as outcomes due to limited cases in this age group.

**Table S6** Sensitivity analysis of factors associated with low appendicular circumferences in participants without cardiometabolic disorders, cancer, or musculoskeletal diseases.

|  | **Low calf circumference** | | | | **Low thigh circumference** | | | | **Low upper arm circumference** | | | | **Low forearm circumference** | | | |
| --- | --- | --- | --- | --- | --- | --- | --- | --- | --- | --- | --- | --- | --- | --- | --- | --- |
|  | OR | 95% CI | | *p* | OR | 95% CI | | *p* | OR | 95% CI | | *p* | OR | 95% CI | | *p* |
| **Urban** | Ref | NA | | NA | Ref | NA | | NA | Ref | NA | | NA | Ref | NA | | NA |
| **Rural** | 1.457 | 1.108 | 1.917 | 0.007 | 1.126 | 0.832 | 1.525 | 0.442 | 0.558 | 0.410 | 0.760 | <0.001 | 0.576 | 0.428 | 0.776 | <0.001 |
| **Education** |  |  |  |  |  |  |  |  |  |  |  |  |  |  |  |  |
| Illiterate | 2.001 | 1.323 | 3.025 | 0.001 | 1.414 | 0.895 | 2.235 | 0.138 | 1.172 | 0.744 | 1.848 | 0.494 | 0.733 | 0.466 | 1.152 | 0.178 |
| Elementary school | 1.716 | 1.232 | 2.390 | 0.001 | 1.430 | 0.997 | 2.052 | 0.052 | 1.173 | 0.834 | 1.650 | 0.360 | 0.946 | 0.680 | 1.315 | 0.740 |
| High school | 1.076 | 0.765 | 1.512 | 0.674 | 1.064 | 0.746 | 1.517 | 0.732 | 0.819 | 0.586 | 1.143 | 0.240 | 0.934 | 0.683 | 1.277 | 0.667 |
| College or above | Ref | NA | | NA | Ref | NA | | NA | Ref | NA | | NA | Ref | NA | | NA |
| BMI | 0.447 | 0.402 | 0.497 | <0.001 | 0.492 | 0.438 | 0.553 | <0.001 | 0.564 | 0.504 | 0.630 | <0.001 | 0.670 | 0.603 | 0.743 | <0.001 |
| %BF | 1.076 | 1.040 | 1.112 | <0.001 | 0.933 | 0.900 | 0.967 | <0.001 | 0.917 | 0.885 | 0.950 | <0.001 | 0.917 | 0.885 | 0.950 | <0.001 |
| Muscle mass | 0.986 | 0.953 | 1.020 | 0.423 | 0.896 | 0.862 | 0.931 | <0.001 | 0.890 | 0.857 | 0.924 | <0.001 | 0.856 | 0.824 | 0.889 | <0.001 |
| **Smoking status** |  |  |  |  |  |  |  |  |  |  |  |  |  |  |  |  |
| Never | Ref | NA | | NA | Ref | NA | | Ref | Ref | NA | | NA | Ref | NA | | NA |
| Former | 1.943 | 1.090 | 3.465 | 0.024 | 1.756 | 0.948 | 3.256 | 0.074 | 1.338 | 0.712 | 2.515 | 0.366 | 3.315 | 1.861 | 5.907 | <0.001 |
| Current | 2.549 | 1.751 | 3.711 | <0.001 | 2.026 | 1.352 | 3.037 | 0.001 | 1.893 | 1.263 | 2.838 | 0.002 | 3.142 | 2.092 | 4.719 | <0.001 |
| **Alcohol drinking** |  |  |  |  |  |  |  |  |  |  |  |  |  |  |  |  |
| Never | Ref | NA | | NA | Ref | NA | | Ref | Ref | NA | | NA | Ref | NA | | NA |
| Former | 1.019 | 0.522 | 1.992 | 0.955 | 0.821 | 0.395 | 1.706 | 0.596 | 0.872 | 0.412 | 1.845 | 0.721 | 0.672 | 0.328 | 1.376 | 0.277 |
| Current | 1.337 | 0.971 | 1.840 | 0.075 | 1.199 | 0.850 | 1.692 | 0.301 | 1.368 | 0.984 | 1.901 | 0.062 | 0.879 | 0.629 | 1.229 | 0.451 |
| **Physical exercise** |  |  |  |  |  |  |  |  |  |  |  |  |  |  |  |  |
| 5~7 days per week | Ref | NA | | NA | Ref | NA | | Ref | Ref | NA | | NA | Ref | NA | | NA |
| 3~4 days per week | 0.981 | 0.638 | 1.510 | 0.932 | 0.924 | 0.576 | 1.481 | 0.742 | 0.792 | 0.509 | 1.233 | 0.302 | 0.622 | 0.395 | 0.980 | 0.041 |
| 1~2 days per week | 0.993 | 0.648 | 1.523 | 0.976 | 1.137 | 0.727 | 1.779 | 0.573 | 0.821 | 0.528 | 1.276 | 0.380 | 0.892 | 0.589 | 1.351 | 0.588 |
| ≤ 3 days per month | 1.029 | 0.650 | 1.628 | 0.904 | 0.714 | 0.418 | 1.219 | 0.217 | 0.972 | 0.607 | 1.558 | 0.908 | 0.703 | 0.433 | 1.141 | 0.154 |
| Never | 1.345 | 1.026 | 1.763 | 0.032 | 1.189 | 0.882 | 1.604 | 0.256 | 0.887 | 0.659 | 1.193 | 0.427 | 0.961 | 0.725 | 1.273 | 0.781 |
| HTN | 1.326 | 1.007 | 1.747 | 0.044 | 1.500 | 1.107 | 2.033 | 0.009 | 1.002 | 0.734 | 1.367 | 0.990 | 1.318 | 0.989 | 1.755 | 0.060 |
| DM | 1.652 | 1.137 | 2.398 | 0.008 | 3.686 | 2.542 | 5.345 | <0.001 | 1.615 | 1.062 | 2.457 | 0.025 | 0.781 | 0.494 | 1.235 | 0.290 |
| HUA | 1.340 | 0.990 | 1.814 | 0.058 | 1.312 | 0.931 | 1.847 | 0.120 | 1.250 | 0.893 | 1.750 | 0.194 | 1.229 | 0.895 | 1.688 | 0.203 |
| Low HDL | 1.223 | 0.789 | 1.895 | 0.369 | 1.879 | 1.188 | 2.973 | 0.007 | 0.881 | 0.518 | 1.500 | 0.642 | 1.169 | 0.735 | 1.857 | 0.510 |
| High non-HDL-C | 1.350 | 0.987 | 1.846 | 0.060 | 1.524 | 1.075 | 2.161 | 0.018 | 1.222 | 0.859 | 1.739 | 0.265 | 1.121 | 0.803 | 1.565 | 0.503 |

HTN: hypertension; DM: diabetes mellitus; HUA: hyperuricemia; HDL-C: high density lipoprotein cholesterol; OR: odds ratio; CI: confidence interval.

**
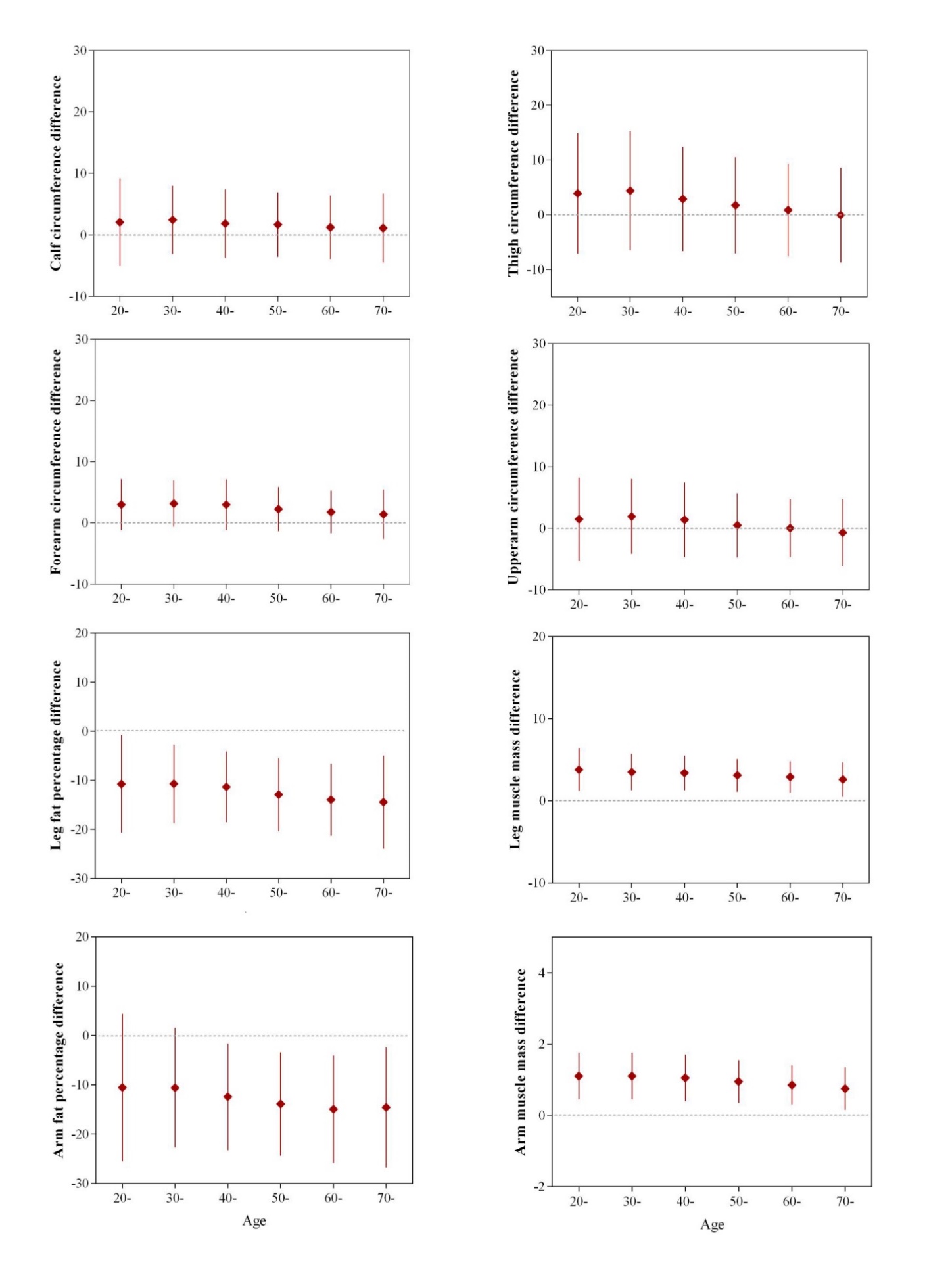
****Figure S1** Comparison of appendicular circumferences between age-matched males and females.


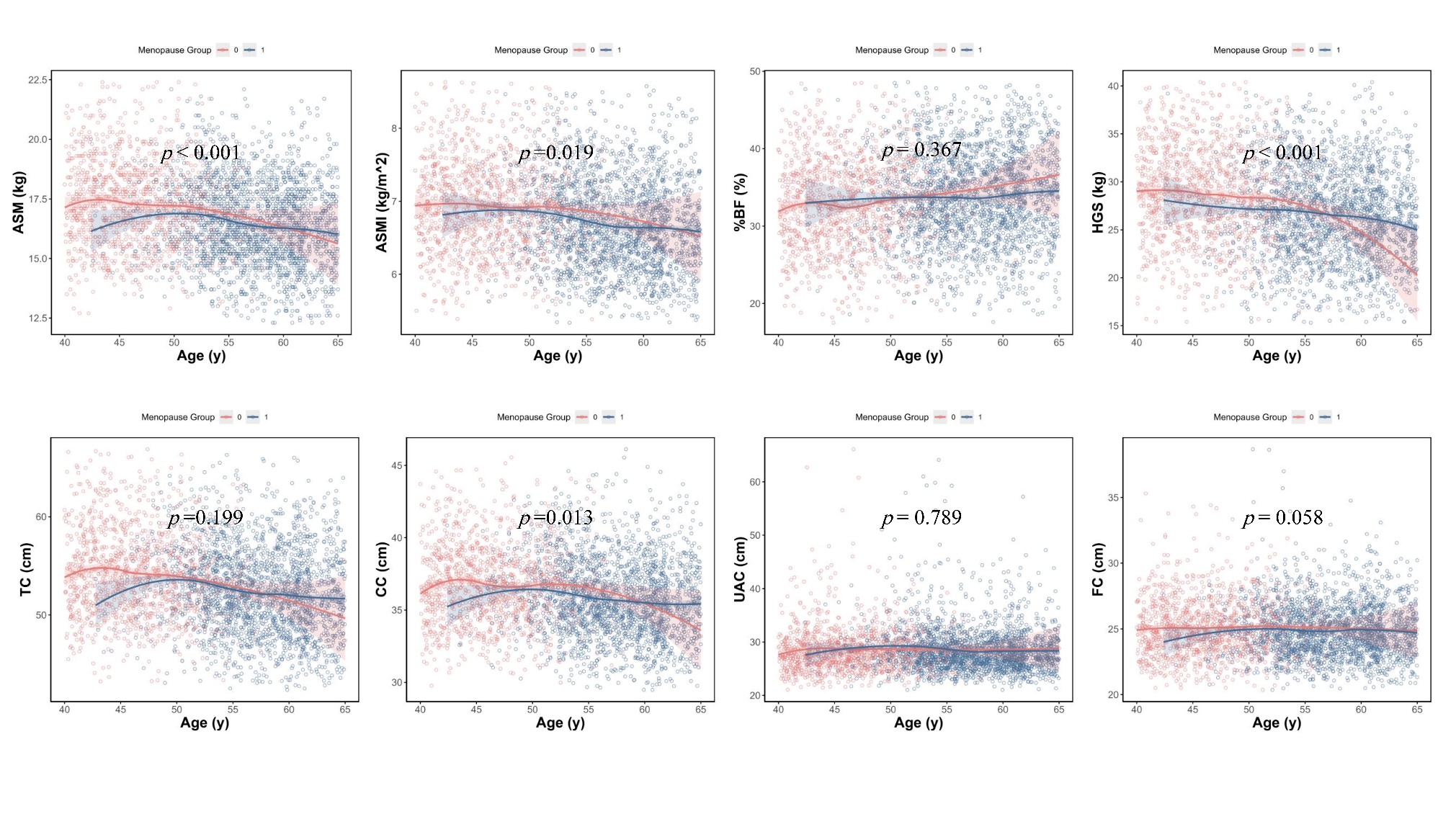
**Figure S2** Comparison of appendicular circumferences, muscle mass, and strength between age-matched postmenopausal and premenopausal women. Menopause status: 0 = premenopause, 1 = postmenopause. ASM: appendicular skeletal muscle mass; ASMI: appendicular skeletal muscle mass index; %BF: body fat percentage; CC: calf circumference; FC: forearm circumference; TC: thigh circumference; UAC: upper arm circumference.
